# BgNet: Classification of benign and malignant tumors with MRI multi-plane attention learning

**DOI:** 10.3389/fonc.2022.971871

**Published:** 2022-10-27

**Authors:** Hong Liu, Meng-Lei Jiao, Xiao-Ying Xing, Han-Qiang Ou-Yang, Yuan Yuan, Jian-Fang Liu, Yuan Li, Chun-Jie Wang, Ning Lang, Yue-Liang Qian, Liang Jiang, Hui-Shu Yuan, Xiang-Dong Wang

**Affiliations:** ^1^ Beijing Key Laboratory of Mobile Computing and Pervasive Device, Institute of Computing Technology, Chinese Academy of Sciences, Beijing, China; ^2^ University of Chinese Academy of Sciences, Beijing, China; ^3^ Department of Radiology, Peking University Third Hospital, Beijing, China; ^4^ Department of Orthopaedics, Peking University Third Hospital, Beijing, China; ^5^ Engineering Research Center of Bone and Joint Precision Medicine, Beijing, China; ^6^ Beijing Key Laboratory of Spinal Disease Research, Beijing, China

**Keywords:** tumor classification, deep learning, multi-plane fusion, benign, malignant

## Abstract

**Objectives:**

To propose a deep learning-based classification framework, which can carry out patient-level benign and malignant tumors classification according to the patient’s multi-plane images and clinical information.

**Methods:**

A total of 430 cases of spinal tumor, including axial and sagittal plane images by MRI, of which 297 cases for training (14072 images), and 133 cases for testing (6161 images) were included. Based on the bipartite graph and attention learning, this study proposed a multi-plane attention learning framework, BgNet, for benign and malignant tumor diagnosis. In a bipartite graph structure, the tumor area in each plane is used as the vertex of the graph, and the matching between different planes is used as the edge of the graph. The tumor areas from different plane images are spliced at the input layer. And based on the convolutional neural network ResNet and visual attention learning model Swin-Transformer, this study proposed a feature fusion model named ResNetST for combining both global and local information to extract the correlation features of multiple planes. The proposed BgNet consists of five modules including a multi-plane fusion module based on the bipartite graph, input layer fusion module, feature layer fusion module, decision layer fusion module, and output module. These modules are respectively used for multi-level fusion of patient multi-plane image data to realize the comprehensive diagnosis of benign and malignant tumors at the patient level.

**Results:**

The accuracy (ACC: 79.7%) of the proposed BgNet with multi-plane was higher than that with a single plane, and higher than or equal to the four doctors’ ACC (D1: 70.7%, p=0.219; D2: 54.1%, p<0.005; D3: 79.7%, p=0.006; D4: 72.9%, p=0.178). Moreover, the diagnostic accuracy and speed of doctors can be further improved with the aid of BgNet, the ACC of D1, D2, D3, and D4 improved by 4.5%, 21.8%, 0.8%, and 3.8%, respectively.

**Conclusions:**

The proposed deep learning framework BgNet can classify benign and malignant tumors effectively, and can help doctors improve their diagnostic efficiency and accuracy. The code is available at https://github.com/research-med/BgNet.

## Introduction

The tumor is one of the main causes of human health problems. According to the statistics of the World Health Organization, millions of people die of cancer every year. Tumors can be divided into benign and malignant. Benign tumors may cause local destruction and even invasive growth of other surrounding tissues, if not detected early. Malignant tumors may cause systemic multisystem metastasis and threaten life. The diagnosis of benign and malignant tumors through patient image data, such as X-ray, CT, or MRI, in the early stage, can guide the formulation of the clinical treatment plan and have important clinical significance.

With the development of computer technology, the use of artificial intelligence (AI) technology for medical image-aided diagnosis has increasingly attracted attention. Traditional image analysis methods generally extract manually designed features and then use support vector machines (1), clustering, decision trees, or artificial neural networks (2) for classification. However, this effect is not ideal in the face of complex data. In recent years, an increasing number of researchers have used deep learning methods from natural images to medical images to assist doctors in diagnosis. The deep learning model can automatically extract and classify the multilevel deep features of the images. Using AI technology to classify benign and malignant tumors in the early stage can help doctors formulate corresponding treatment plans for patients in time and prevent deterioration of the disease, which have very important clinical and research values.

Professionally trained doctors can easily locate tumor regions in images, but it is difficult to classify benign or malignant for the complex appearance of tumors. Some tumors usually have the characteristics of high heterogeneity, diverse location, unclear boundary, and unclear visual characteristics. In the clinic, radiologists usually need to observe multiple plane images, such as axial, sagittal, or coronal planes for comprehensive judgment. Existing AI analysis of medical images can be divided into two categories according to the data modality. One is single-modality data, which is used to analyze medical images through data processing and model improvement (3–6). Due to the single source of single-modality data, it may have limitations in some tasks. The other is multi-modal data, which carries out the corresponding tasks by constructing multi-modal models including cross-modal analysis, such as CT and MRI (7), MRI and ultrasound (8), MRI and PET (9), and also different sequences of CT (10, 11) or MRI (12). While the aforementioned methods are focused on the same single planes, such as axial or sagittal. The same tumor can demonstrate various shapes in different planes, which has certain internal relevance and need to be explored from multi-plane image data. In addition, the patient’s clinical information has a great reference value for diagnosis. Existing methods lack the effective combination of different planes or even patients’ clinical information.

To solve the aforementioned problems, this study proposed a multi-plane fusion framework named BgNet for tumor benign and malignant diagnosis at the patient level, which used a bipartite graph to splice different plane images at the input layer, and an attention mechanism model to carry out feature association mining and multilevel fusion on different plane images. BgNet can integrate different plane images and clinical age information of the same patient for patient-level tumor benign and malignant diagnosis. In addition, with the help of BgNet, the diagnostic accuracy and speed of doctors can be improved. Compared with other methods for processing single frame images, the main innovation of this paper is to apply bipartite graph to medical images, and use the proposed ResNetST model for multi-plane fusion, which can make the model more comprehensive in observing patients’ images.

## Materials and methods

### Dataset

**Figure 1:**
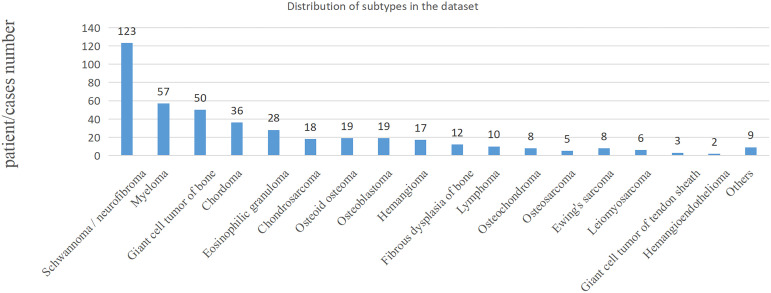
Distribution of subtypes in the dataset.

In the clinic, bone tumors are usually diagnosed by observing multiple planes of medical images. The performance of the proposed method was evaluated on the classification task of benign and malignant spine tumors, which is challenging due to the complex appearance of images arising from tumor heterogeneity and varying locations. This study collected the MRI data of primary spinal tumors from 2006 to 2019 from the participating hospital, and it was approved by the Medical Science Research Ethics Committee review board. A total of 430 patients or cases with both axial and sagittal sequences and pathological reports were selected for experiments. Radiologists marked the tumor region using a rectangular box, which was checked by each of them for reliability. The benign and malignant classifications of these regions were based on the patients’ pathological reports. Detailed information of the dataset is shown in **Table 1**, and the distribution of subtypes in the dataset is shown in **Figure 1**; the age distribution was large, some tumors were multiple, and the locations of the tumor varied, such as cervical, thoracic, or lumbar vertebrae, which are complex and challenging for AI model.

**Table 1:** The specific information of the training set and testing set.

		Training Set (n = 297)	Testing Set (n = 133)
Age (years, mean±SD)	–	40.2± 20.5	38.8± 19.1
Gender	Female	152 (51.2%)	48 (36.1%)
	Male	145 (48.8%)	85 (63.9%)
Slice thickness	–	3.0mm~8.0mm	3.0mm~6.0mm
Location	cervical vertebrae	167	72
	thoracic vertebrae	89	48
	lumbar vertebrae	73	27
	sacral vertebrae	15	5
Number of Sequences	Axial	491	230
	Sagittal	1089	491
Number of Images	Axial	4049	1597
	Sagittal	10023	4564
Labeled Images	–	14072	6161

SD, standard deviation.

### Method

**Figure 2:**
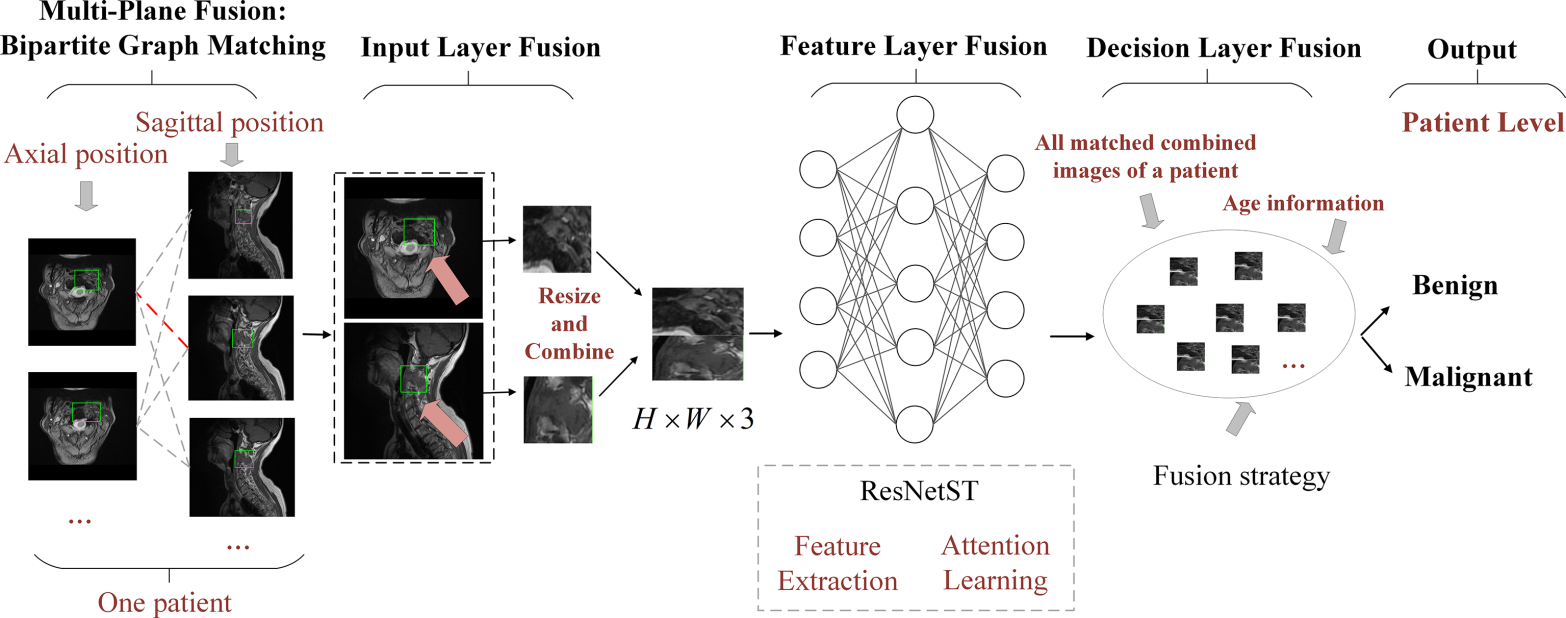
The framework of BgNet is divided into five modules, including a multi-plane fusion module based on the bipartite graph, an input layer fusion module, a feature layer fusion module, a decision layer fusion module, and an output module. In the first part, the matching pairs between different planes are constructed through the bipartite graph. In the second part, the tumor regions on different planes represented by each matching pair are fused in the input layer. In the third part, the proposed model named ResNetST is used to extract and fuse the features of different planes. Finally, all the matching edges between the different planes of the patients are integrated through a trusted edge set screening strategy to obtain the final patient-level classification results.

Most existing AI methods are based on single plane analysis. In the clinic, radiologists usually need to observe multiple plane images and sequences of the same patient for comprehensive judgment. Inspired by this processing, this study proposes a comprehensive diagnostic AI framework, BgNet, which used a bipartite graph to fuse the data of the two planes, the tumor area in each plane is used as the vertex of the graph, and the matching between different planes is used as the edge of the graph. By integrating all edges in the bipartite graph, the patient-level diagnosis results of benign and malignant tumors can be obtained. The framework of BgNet can be seen in **Figure 2**, which consists of five parts. In the first part, the matching pairs between different planes are constructed through the bipartite graph. In the second part, the tumor areas on different planes represented by each matching pair are fused in the input layer. The tumor areas of axial and sagittal images were scaled, and combined up and down to form a single image in the input layer. In the third part, the proposed model named ResNetST to extract and fuse the features of different planes from the input layer, with the convolutional neural network ResNet50 (13) as the feature extraction module and the Swin-Transformer (14) model based on the attention learning as the global feature fusion module. Finally, all matching edges between different planes of patients are integrated through a trusted edge-set screening strategy to obtain the final patient-level diagnosis results. The patients’ biopsy-confirmed labels were used to train and test the model. This paper focuses on the fusion of different planes and the same plane can contain different sequences, e.g. T1, T2.

#### Multi-plane fusion module based on the bipartite graph

Bipartite graph is a special model in the graph theory (15). Let *G*(*V*, *E*) be an undirected graph. If vertex *v* can be divided into two disjoint subsets *A* and *B* , and the two vertices *i* and *j* associated with each edge *e*(*i*, *j*) in the graph belong to these two different vertex sets (*i*∈*A*,*j*∈*B*) , then the graph *G* is called a bipartite graph.

Referring to the radiologists’ reading process, we proposed a multi-plane fusion strategy based on a bipartite graph. First, we constructed a bipartite graph, as shown in **Figure 2**. Here, we consider MRI axial and sagittal images and divide images of the same patient into two plane sets. The axial images were set *A* , and the sagittal images were set *B* . Each image in the set was used as the vertex to connect the graphs to form a complete bipartite graph. These connections are called matching edges. Assuming that there are *n* images in set *A* and *m* images in set *B*, we obtain *n*×*m* matching edges between *A* and *B* . The edges in the bipartite graph will be fused in the input layer fusion phase described below, and then the common features will be extracted using the deep learning model.

In the training stage, the edge *e*(*i*,*j*) between set *A* and set *B* was randomly activated. The model takes the activated edge as the input to perform the input layer fusion and feature layer fusion and calculates the loss function and backpropagation of the gradient. In the test stage, all edges of *E* in sets *A* and set *B* were activated. The model takes all edges as inputs to perform input layer fusion, feature layer fusion, and decision layer fusion.

#### Input layer fusion module

The input layer fusion module is shown in **Figure 2**. Two vertices *i* and *j* of the activated edge *e*(*i*,*j*) , which are a frame of the axial image and a frame of the sagittal image, are fused in the input layer. The tumor areas of axial and sagittal images were scaled, combined up and down to form a single image, in which the axial is above and sagittal is below as shown in **Figure 2**, and resized to form a 224×224×3 image. Then, the feature-layer fusion model was used to extract the common features.

Before sending the image to the feature-layer fusion model, a series of data augmentation techniques will be done to prevent the model from overfitting, which will be introduced next. In the training stage, we did not extract the tumor area marked by the doctors strictly, but expanded 40 to 60 pixels around the marked tumor area, so that the model can also learn the information around the tumor. In addition, we randomly flipped the tumor area, including the up and down direction, left and right direction. In the stage of training and testing, we used $\frac{x_{i}-\mu }{\sigma }$ to standardize the image, where *μ* is the mean value of all pixels of the image, and *σ* is the standard deviation of all pixels of the image.

#### Feature layer fusion module

**Figure 3:**
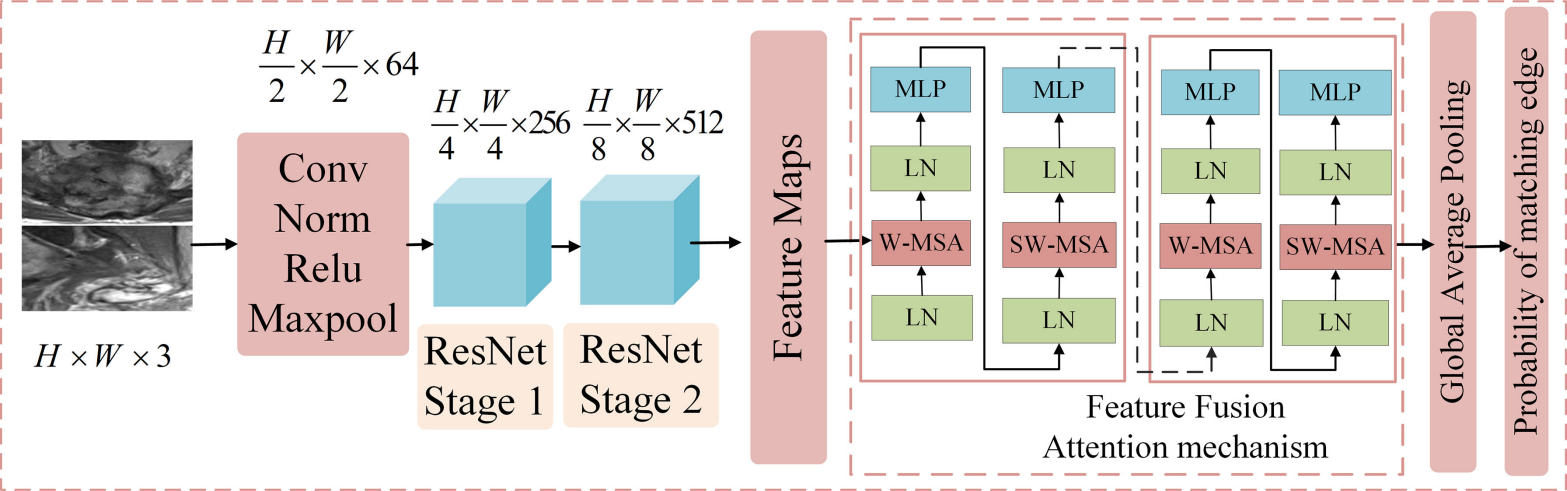
The structure of the feature layer fusion module. It includes five parts. The first part contains Conv, Norm, Relu, and max pool operations to reduce the dimension of the input image and reduce the computational complexity. The second part is ResNet Stage 1, which performs feature extraction at 1/4 the resolution of the image. The third part is ResNet Stage 2, which performs feature extraction at 1/8 the resolution of the image. The fourth part is the feature fusion module, which uses the attention mechanism to correlate the extracted features globally and extracts deeper features. The fifth part includes the global average pooling and output, which employs fully connected networks as a classifier to obtain the benign and malignant probabilities of these edges.

Convolutional neural network (CNN) can adaptively learn the features and spatial hierarchy of images (16), and the attention mechanism can correlate the global features well (17), Based on the above technologies, we proposed ResNetST as the feature layer fusion model by feature extraction and attention learning, and the network structure is shown in **Figure 3**, including five parts. The first part contains conv, norm, relu, and maxpool operations to reduce the dimension of the input image and reduce the computational complexity. The second part is ResNet Stage 1, which performs feature extraction at 1/4 resolution of the image. The third part is ResNet Stage 2, which performs feature extraction at 1/8 resolution of the image. The fourth part is the feature fusion module, which uses the attention mechanism to correlate the extracted features globally and extracts deeper features. The fifth part includes the global average pooling and output, which uses fully connected networks as classifiers to get the benign and malignant probabilities of these edges. In the training stage, cross-entropy was used to calculate the loss, and the BP algorithm (18) was used for gradient backpropagation and model parameter updating. In the testing stage, the benign and malignant probabilities of the edge are directly output.

#### Decision layer fusion module and output module

Decision-layer fusion was only used in the testing (or inference) stage. All edges of the above bipartite graph are activated and predict these matching edges one by one to obtain the probability that they are benign or malignant. For the same patient, set *C* is obtained in the decision layer fusion module, in which each element is the benign and malignant probability of the corresponding matching edge. The purpose of decision layer fusion is to judge the overall benign and malignant category of the patient according to set *C*.

In clinical practice, the age information of patients has a significant reference for diagnosis. First, we obtained the relationship set *K* between the patient’s age and the probability of benign and malignant tumors according to the statistical information of all patients in the training set. Each element in set *C* and the probability of benign and malignant tumors at the patient’s current age are weighted and summed, and the weights are *λ*_1_ and *λ*_2_ , wherein *C*_*i*_=*λ*_1_×*C*_*i*_+*λ*_2_×*K*_*i*_ . Then, each element in set *C* is sorted from large to small, and the *topk* elements with the largest probability are taken out to form a set *S* , that is, the *topk* matching edges that the model considers to be more accurate in prediction. Then, taking *T* as the threshold, the matching edge with probability greater than *T* in set *S* will be judged as malignant, less than *T* will be judged as benign, and equal to *T* will be discarded. Next, we obtain the number *q* of benign matching edges and the number *w* of malignant matching edges in the set *S* , and calculate the probability $p_{b}=\frac{q}{q+w}$ for predicting benign tumors and $p_{m}=\frac{w}{q+w}$ for predicting malignant tumors for the current patient. If *p*_*b*_>*p*_*m*_ , the model predicts that the current patient has a benign tumor, and if *p*_*b*_≤*p*_*m*_ , the model predicts the current patient as having a malignancy.

### Experimental design

In our experiments, MRI axial and sagittal images of 430 cases with primary spine tumors from 2006 to 2019 from the participating hospital were used, with 297 cases included for training and 133 cases for testing, and all cases were approved by the Medical Science Research Ethics Committee review board. We design the following experiments to verify the effectiveness of the proposed multi-plane fusion framework.

Firstly, we conducted experiments based on a single plane, such as using only axial or sagittal images. That is, we use ResNetST mentioned above as the classification model and use the patient’s axial or sagittal image data for training and testing. In the test stage, according to all the axial or sagittal data of the patient, we will get set *C* , where each element is the probability that each image is benign or malignant. Like the decision layer fusion mentioned above, we can get the patient-level benign and malignant diagnosis results through set *C* .

Secondly, we conduct mixed training on an axial plane and sagittal plane and finally make decision layer fusion. This is, we also use ResNetST mentioned above as the classification model, but mixed the axial and sagittal image data for training and testing. The mean of “mixed” is that during training and testing, the axial and sagittal planes are still single frame independent images. If the patient has *n* axial images and *m* sagittal images, the set *C* containing *n*+*m* elements will be obtained in the test stage. Similarly, patient-level fusion is performed according to set *C* to obtain the overall benign and malignant diagnosis result of the patient.

Third, we used multi-plane fusion based on a bipartite graph. That is, different from the previous two experiments, we matched the axial and sagittal images of patients and then fused the input layer and feature layer. Assuming that the patient has *n* axial images and *m* sagittal images, in the test stage, a set C containing *n*×*m* elements will be obtained. Similarly, the patient-level diagnosis results are obtained according to set *C* .

Finally, we evaluated the performance of referring to age information based on the first three experiments. In our experiments, the proposed ResNetST was better than the original ResNet (13) and Swin-Transformer (14) as the feature-layer fusion module. ResNetST can combine the advantages of ResNet and Swin-Transformer for this task. All experiments used ResNetST as a feature layer fusion model, but this does not mean that in BgNet, the feature layer fusion model must be ResNetST, it can be changed according to different tasks.

To ensure the fairness of comparison, all the above experiments use the same feature layer fusion model, decision layer fusion module, and super parameters except for the different data (single-plane or multi-plane). The results of AI methods were also compared with four doctors, including one spine surgeon (D2: 8 years experience) and three radiologists (D1: 3 years experience; D3: 11 years experience; D4: 18 years experience). Four doctors independently predicted benign and malignant tumors by checking axial and sagittal MRI images with age information on the testing set. All doctors had trained in neuroradiology or musculoskeletal fellowship. In addition, we also evaluated the results of doctors assisted by an artificial intelligence model.

### Metrics

In this study, samples of malignant tumors were considered positive samples, and the area under the curve (AUC), accuracy (ACC), sensitivity (SE), specificity (SP), and average time spent to predict each case were used as evaluation metrics. AUC is obtained by drawing a ROC curve for the probability of benign and malignant tumors diagnosed by each patient according to the model and taking the area under the curve. Since the best classification threshold of the model cannot be known in advance, in order to be fair, we take 0.5 as the classification threshold *T* to calculate ACC, SE, and SP. Then, we get the confusion matrix including TP, FP, TN, and FN. The calculation method of ACC, SE, and SP is as shown in equations (1), (2), and (3). We take the average time of each patient in the model and doctor diagnostic test set as the metrics of diagnostic efficiency. It should be noted again that we calculate all the above metrics based on the patient-level diagnostic results obtained from all the patient’s image data.


(1) $$ACC=\frac{TP+TN}{TP+TN+FP+FN}$$

(2) $$SE=\frac{TP}{TP+FN}$$

(3) $$SP=\frac{TN}{FP+TN}$$

### Training setting

We evaluated our artificial intelligence model using the testing set containing 133 cases or patients. In the training stage, all methods used the same super parameters, with a learning rate of 0.0002. Stochastic gradient descent was used as the optimizer (19) and the number of iterations was 20 epochs. The super parameters of ResNetST are the same as ResNet50 and Swin-Transformer Tiny. The ResNet Stage 1 and Stage 2 in ResNetST are initialized using the pre-trained model on ImageNet. All methods use four 12G 1080ti GPUs for distributed training. In the decision layer fusion, $\lambda_{1}=0.6,\;\lambda_{2}=0.4,\;T=0.5,\;topk=\text{max}\left(300,\frac{size\left(C\right)}{2}\right)$ . All experiments used the same decision layer fusion method to obtain the patient-level results.

## Results

### Comparison of the different methods

In **Table 2**, the AUC of the model with only reference to the sagittal image (Sag: 73.1%) is 1.0% higher than that of the axial image (Axi: 72.1%). The AUC with mixed axial and sagittal images (Axi_Sag: 76.7%) is 4.6% and 3.6% higher than that of single plane Axi and Sag. Based on the BG, the AUC of BG_Axi_Sag (81.8%) was further improved by 9.7%, 8.7%, and 5.1% compared to Axi, Sag, and Axi_Sag, respectively.

**Table 2:** Results of the different methods in the classification of benign and malignant tumors based on the primary spinal tumors dataset (95% CI).

Age	Plane	Method	AUC (%)	ACC (%)	SE (%)	SP (%)
✘	single	Axi	72.1±7.6	68.4±7.9	61.2±8.3	72.6±7.6
		Sag	73.1±7.5	67.7±7.9	49.0±8.5	78.6±7.0
	multi	Axi_Sag	76.7±7.2	70.7±7.7	61.2±8.3	76.2±7.2
		**BG_Axi_Sag**	**81.8**±6.6	**70.7**±7.7	**87.8**±5.6	60.7±8.3
✔	single	Axi_Age	71.3±7.7	69.2±7.8	59.2±8.4	75.0±7.4
		Sag_Age	75.4±7.3	65.4±8.1	44.9±8.5	77.4±7.1
	multi	Axi_Sag_Age	79.0±6.9	72.2±7.6	59.2±8.4	79.8±6.8
		**BG_Axi_Sag_Age (BgNet)**	**84.3**±6.2	**79.7**±6.8	**91.8**±4.7	72.6±7.6

**Axi** represents the axial images. Sag represents the sagittal images. BG represents the bipartite image-fusion strategy. Age represents a reference to age. CI, confidence interval; AUC, area under the curve; ACC, accuracy; SE, sensitivity; SP, specificity. Bold indicates the result with the best result.

After referring to the age information, the AUC of all the AI models improved. The AUC of Sag_Age was 4.1% higher than that of Axi_Age. The AUC of the mixed axial and sagittal images was 7.7% and 3.6% higher than that of Axi_Age and Sag_Age. BgNet can obtain the highest AUC (84.3%) and ACC (79.7%) after referencing the age. The AUC of BgNet is improved by 2.5% to 13.0%, and the ACC is improved by 7.5% to 14.3% with age information compared to the other methods.

### Comparison of the model and doctors

In the case of reference the age, the comparison results with doctors are shown in **Table 3**. The ACC of BgNet was equal to D3 and was 9.0%, 25.6%, and 6.8% higher than those of D1, D2, and D4, respectively. The SE of BgNet was 2.0%, 22.4%, and 20.4% higher than those of D1, D3, and D4, respectively. Although the SE of D2 (95.9%) was higher than that of BgNet, the corresponding SP (29.8%) was low. The SP of BgNet (72.6%) was 13.1% and 42.8% higher than that of D1 and D2, respectively, which approached D4 (73.8%). Although the SP of D3 (85.7%) was higher than that of BgNet, its SE (69.4%) was low. The ACC of D3 (79.7%) was equal to that of BgNet; however, the average time for predicting a patient was 74.9 s, while it was only 0.7 s for BgNet.

**Table 3:** Comparison of the results between the model and the doctors (95% CI).

Method		ACC (%)	SE (%)	SP (%)	Avg. Time (s)	p-value
Doctors	D1	70.7±7.7	89.8±5.1	59.5±8.3	29.5	p=0.219
	D2	54.1±8.5	95.9±3.4	29.8±7.8	18.8	p<0.005
	D3	79.7±6.8	69.4±7.8	85.7±5.9	74.9	p=0.006
	D4	72.9±7.6	71.4±7.7	73.8±7.5	31.4	p=0.178
**AI Model**	**BgNet**	**79.7**±6.8	**91.8**±4.7	**72.6**±7.6	**0.7**	–

Three radiologists D1, D3 and D4 and one spine surgeon D2. D1: 3 years’ experience; D2: 8 years’ experience; D3: 11 years’ experience; D4: 18 years’ experience CI, confidence interval; AUC, area under the curve; ACC, accuracy; SE, sensitivity; SP, specificity. Bold indicates the result with the best result.

### Comparison of the different vertebral locations

**Figure 4:**
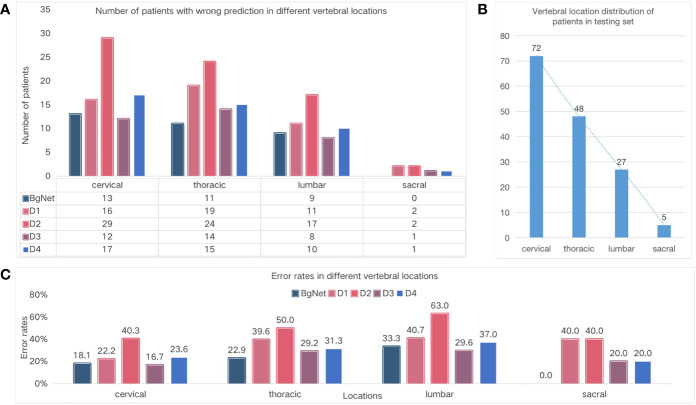
Comparison of the different vertebral locations. **(A)** Number of cases with wrong prediction in different vertebral locations. **(B)** Vertebral location of the distribution of cases in the testing set. **(C)** Error rates in the different locations.

To specifically analyze the results of BgNet and doctors, we counted the number of patients with incorrect prediction and error rates in different vertebral locations, as well as the distribution of vertebral locations in the testing set, as shown in **Figure 4**.

The number of cases with incorrect prediction and error rates by BgNet in each location was lower than those of the doctors. As shown in **Figure 4A**, BgNet, D2, and D4 had the largest incorrect predictions at the cervical vertebra, and D1 and D3 had the largest number in the thoracic vertebra, while both BgNet and doctors had the lowest number in the sacral vertebra. By observing the number distribution in different vertebral locations in **Figure 4B**, the number of cases in the cervical and thoracic vertebrae is large, and the misprediction trend of BgNet and doctors is consistent with the location distribution.

However, as shown in **Figure 4C**, the error rate trend of BgNet and doctors is different from that of **Figure 4B**. For example, most doctors and BgNet have a lower error rate in the cervical vertebrae and the highest error rate in the lumbar vertebrae. The reason for this phenomenon is that both BgNet and doctors need to accumulate experience from a large number of cases. The more cases, the richer the experience, and the lower the error rate. Our testing set generally reflects the actual distribution of patients in cooperative hospitals. With the largest number of cases in the cervical vertebra, the number of patients with incorrect predictions may be higher, while the error rate is lower.

### Comparison of the different age groups

**Figure 5:**
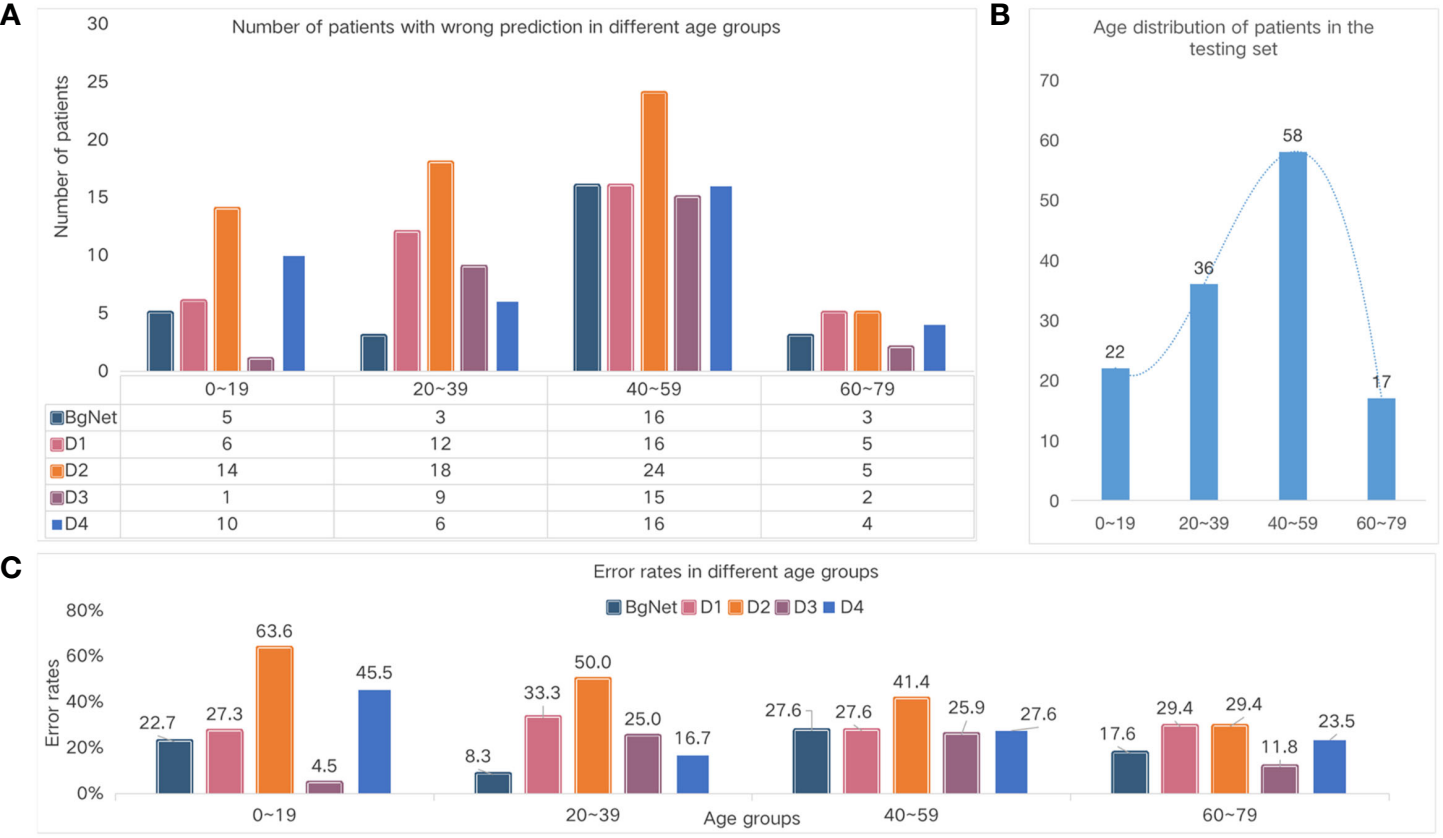
Comparison of the different age groups. **(A)** Number of cases with the wrong prediction in different age groups. **(B)** The age distribution of the cases in the test set. **(C)** Error rates in the different age groups.

In addition to vertebral locations, we also specifically analyzed the results according to different age groups, as shown in **Figure 5**. From **Figure 5A** and **Figure 5C**, the number of cases with incorrect prediction and error rate by BgNet in each age group is lower than most doctors, and the error rate in the two age groups of 20–39 and 60–79 is lower than that of the other two age groups.

The number of wrong cases in the 40–59 age group was the largest for both doctors and BgNet. The 40–59 age group included 31 cases of cervical vertebrae, 17 cases of thoracic vertebrae, nine cases of lumbar vertebrae, and one case of sacral vertebrae. In this age group, the BgNet mispredicted nine cervical cases, five thoracic cases, and five lumbar cases. Overall, the error rate of BgNet in this age group was 29.0%, 29.4%, 55.6%, and 0% for the cervical, thoracic, lumbar, and sacral vertebrae, respectively. Combined with **Figure 4**, it can be seen that the increase in the error rate of the thoracic and lumbar vertebrae caused an increase in the overall error rate in this age group. In addition, the error rate trends of D1 and D2 are relatively consistent, and the error rate of D3 in the age group 0–19 is much lower than that of BgNet, but in the age group 20–39 is much higher than that of BgNet.

### Results of the classification by doctors assisted with the AI model

To evaluate whether the AI model can assist doctors in improving their diagnosis accuracy and efficiency, we again invited those 4 doctors to label the testing cases. Based on MRI data, age information and additional malignant probability of each case given by BgNet model, doctors can make their final comprehensive diagnosis for benign or malignant tumor. The results are shown in **Table 4**, the diagnostic accuracy and speed of all four doctors have been significantly improved. For example, with the highest diagnostic accuracy in **Table 4**, D3’s ACC increased from 79.7% to 80.5%. D4’s ACC increased from 72.9% to 76.7%, and his diagnostic time for each case decreased from 31.4 s to 15.6 s. So with the help of BgNet, doctors can improve their diagnostic accuracy and diagnose twice as fast as before.

**Table 4:** Classification results of the doctors assisted by BgNet.

Doctors	ACC (%)	SE (%)	SP (%)	Avg. Time (s)
D1	75.2+4.5	95.9+6.1	63.1+3.6	17.0-12.5
D2	75.9+21.8	87.8-8.1	69.1+39.3	13.5-5.3
D3	80.5+0.8	89.8+20.4	75.0-10.7	34.3-40.6
D4	76.7+3.8	75.5+4.1	77.4+3.6	15.6-15.8

Based on the MRI data, age information, and additional malignant probability of each case given by the BgNet model, doctors made their final comprehensive classification for benign or malignant tumors. D1, D3, and D4, and one spine surgeon D2. D1: 3 year experience; D2: 8 year experience; D3: 11 year experience; D4: 18 year experience. AI, artificial intelligence; AUC, area under the curve; ACC, accuracy; SE, sensitivity; SP, specificity.

### Heat maps analysis

**Figure 6:**
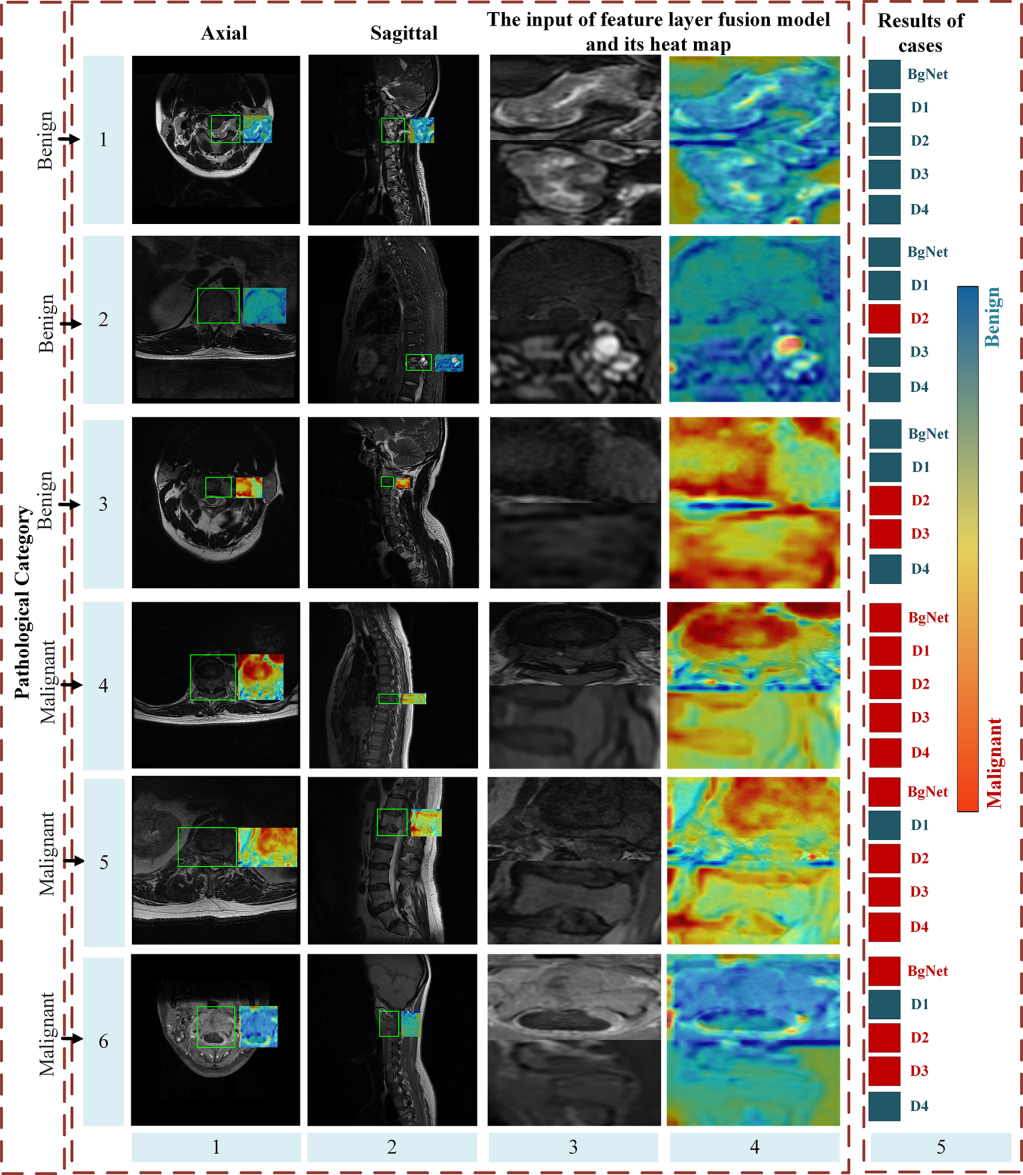
Part heat maps of BgNet. The redder the color, the more inclined the model is to predict it as malignant, and the bluer the color, the more inclined the model is to predict it as benign. The images of each line are from different cases, including three benign and three malignant cases, and the ground truth is listed in the first column. The axial and sagittal images in the left two columns contain a rectangular box marked by doctors. The third column is one of the edges of the bipartite graph used as the input of the feature-layer fusion model. The fifth column shows the classification results of the cases obtained by BgNet from all the edges of the bipartite graph of the case and the results obtained by the four doctors. The blue square indicates that the case was classified as benign, and the red square indicates that it was classified as malignant.

**Figure 6** shows some heat maps of BgNet, which can represent the information focused on the model in the inferencing process. The images of each line in **Figure 6** are from different cases, including three benign and three malignant cases, and the ground truth is listed in the first column. The axial and sagittal images in the left two columns contain a rectangular box marked by a doctor. The third column is one of the edges of the bipartite graph used as the input of the feature-layer fusion model. The fifth column shows the diagnosis results of cases obtained by BgNet from all edges of the bipartite graph of the case and the results obtained by the four doctors. The blue square indicates that the case was diagnosed as benign, and the red square indicates that it was diagnosed as malignant. Heat maps are generated based on Grad-CAM++ (20), which uses a weighted combination of the positive partial derivatives of the last convolutional layer feature maps concerning a specific class score as weights to generate a visual explanation for the corresponding class label. The redder the color, the more inclined the model is to predict it as malignant, and the bluer the color, the more inclined the model is to predict it as benign.

It can be seen that BgNet obtains the correct diagnosis results for these six cases. For cases in lines 1 and 4, both BgNet and doctors make a correct diagnosis. From the heat map of one edge of the bipartite graph represented by these lines, BgNet can correct the response to benign and malignant regions. For the cases in lines 2 and 5, the prediction results of D2 and D1 were incorrect. It can be seen from line 2 that although BgNet focused on a suspected malignant area in the sagittal image, it finally made a correct judgment at the case level from the integration of the axial information, which shows the effectiveness of taking the edge of the bipartite graph as input. As predicted, the BgNet, will not be 100% correct when predicting each edge of the bipartite graph of the case. As shown in lines 3 and 6, BgNet tends to predict the edge represented by line 3 as malignant and the edge represented by line 6 as benign, which is contrary to the pathological results. However, the final diagnosis result for BgNet was correct. This is because, in the constructed bipartite graph, a single edge cannot represent the final diagnosis result. BgNet fuses the results of all edges at the decision layer and finally makes a judgment. In addition, for the cases represented by lines 3 and 6, two doctors made a misjudgment, which shows that the proposed BgNet has better adaptability to complex samples.

## Discussion

Predicting benign and malignant tumors through patient images at an early stage is important for clinical treatment plans. Taking spine tumors as an example, inspired by the doctors’ diagnosis processing by checking multiple plane images, we proposed a novel framework, BgNet, to combine the patient’s axial and sagittal images with a bipartite graph strategy at the input layer. Then, the feature layer fusion model ResNetST fuses the axial and sagittal tumor areas represented by the edges in the bipartite graph at the feature layer. Finally, the results of all edges of the bipartite graph and clinical age information are integrated to obtain the final patient-level diagnosis results for the benign and malignant diagnoses of tumors.

In recent years, deep learning methods (21–23) have gradually become the main methods in the field of pattern recognition and computer vision since 2012. The tumor diagnosis in the medical image refers to the use of a deep learning model, such as ResNet or VGG (24) to extract the features and classify the corresponding image or tumor area. Hong et al. (25) proposed a multi-information fusion framework based on MRI sagittal images, which can integrate a detection model and a sequence classification model for patient-level diagnosis. In recent years, the attention mechanism-based model Transformer (26) can better extract and correlate global information, which can be used for medical imaging.

Multi-modal fusion of medical images has received increasing attention in the fields of medicine and computers, which can be divided into input-level fusion, feature-level fusion, and decision-level fusion (27). At the data level, it can be divided into image fusion of the same modality with different parameters, multi-sequence fusion, cross-modality, multiple planes, images, and clinical information fusion. For input level fusion, Yang et al. (28) created a channel splice for the four different scanning images, T1, T1c, T2, and flair in the input stage, and then sent them to the deep learning model for feature extraction and fusion. For feature-level fusion, Dolz et al. (29) proposed HyperDenseNet, a 3D full convolution neural network based on DenseNet (30), which extends the definition of dense connectivity to the multimodal segmentation model, receiving T1 and T2 sequences of MRI as inputs and achieving great results for brain tumor segmentation. Chen et al. (31) proposed a dual-branch multimodal brain tumor segmentation network, which uses two branches to extract the features of T1 and T2, respectively, and fused them at the end of these branches. Zhou et al. (9) proposed a multi-modality framework based on a deep non-negative matrix factorization model, which can fuse MRI and PET images for the diagnosis of dementia. Zhang et al. (10) proposed a modality-aware mutual learning method, which can fuse the arterial and venous phases of CT images for tumor segmentation. Most methods usually take decision level fusion as a part of the whole method, mainly including majority voting, averaging (32–34), etc. In addition, Reda et al. (35) built an additional classifier, which used the prediction probability of different modal models as input to classify the decision level, and the performance was improved compared with the model with a single modal only. Most of the existing methods are aimed at the fusion of single-plane data, and lack a multi plane fusion method.

In contrast to the above method, we adopted a bipartite graph strategy to directly combine the different plane data, which can be used for further associated feature extraction and also greatly expands the number of training samples for deep learning model training. The attention mechanism in the ResNetST model can further extract the local and associated feature expressions from patient multi-plane data. Age information can improve final diagnosis performance. The experimental results show that the proposed BgNet can approach or exceed four medical experts, and with the help of BgNet, the diagnostic accuracy and speed of doctors have been significantly improved, which has clinical significance in medical image-aided diagnosis. Although we have carried out experiments on spine tumors and achieved certain results, the method is universal and can also be used in the classification of other tumors.

The proposed BgNet still has room for improvement. In the test stage, we used all matching pairs between different planes. For example, an image of the axial corresponds to all sagittal images and this is effective in our experiment. While there may be some matching pairs that ultimately have a significant impact on the patient-level diagnosis. How to select these matching pairs in the early stage when constructing the bipartite graph? In addition, the certain correlation for the tumor areas of cross-modality, such as CT and MRI, will be existed, which should be studied and optimized on the cross-modal data based on our framework in the future.

## Conclusions

Due to the complex data of tumors, it is difficult to accurately diagnose patients only through a single plane. This paper proposed a novel multi-plane fusion framework BgNet, which fuses axial and sagittal MR images through an attention mechanism based on a bipartite graph and makes patient-level diagnosis combined with clinical age information. Experimental results showed that BgNet can efficiently identify benign and malignant tumors, and with the help of the AI model, the diagnostic accuracy and speed of doctors can be significantly improved.

## Data availability statement

The original contributions presented in the study are included in the article/supplementary material. Further inquiries can be directed to the corresponding authors.

## Author contributions

HL and M-LJ processed data, proposed methods, designed experiments, analyzed results, wrote, and modified the manuscript. X-YX, H-QO-Y, YY, J-FL, YL, C-JW, NL, LJ, and H-SY collected original data, labeled tumors, provided clinical suggestions, and reviewed the manuscript. Y-LQ and X-DW gave suggestions on methods. All authors contributed to the article and approved the submitted version.

## Funding

This work was supported by the Beijing Natural Science Foundation (Z190020), National Natural Science Foundation of China (62276250, 82171927, 81971578), Capital's Funds for Health Improvement and Research (2020-4-40916).

## Conflict of interest

The authors declare that the research was conducted in the absence of any commercial or financial relationships that could be construed as a potential conflict of interest.

## Publisher’s note

All claims expressed in this article are solely those of the authors and do not necessarily represent those of their affiliated organizations, or those of the publisher, the editors and the reviewers. Any product that may be evaluated in this article, or claim that may be made by its manufacturer, is not guaranteed or endorsed by the publisher.
